# Localized amyloidosis of the ureter: a case report

**DOI:** 10.1186/s13256-023-04138-y

**Published:** 2023-10-08

**Authors:** Shuntaro Aoki, Takashi Kawahara, Hironao Tajirika, Masato Yasui, Hideyuki Terao, Makoto Funahashi, Kazuhide Makiyama, Hiroji Uemura, Hiroyuki Hayashi, Junichi Ohta

**Affiliations:** 1 Departments of Urology, Yokohama Municipal Citizen’s Hospital, Yokohama, Japan; 2 Departments of Urology, Yokohama City University Medical Center, Yokohama, Japan; 3 Departments of Urology, Yokohama City University Graduate School of Medicine, Yokohama, Japan; 4 Department of Pathology, Yokohama Municipal Citizen’s Hospital, Yokohama, Japan; 5 Departments of Urology and Renal Transplantation, Yokohama City University Medical Center, Yokohama, Japan

**Keywords:** Localized amyloidosis, Ureteral tumor, Amyloidosis

## Abstract

**Background:**

Amyloidosis is a collection of disorders characterized by the extracellular deposition of amyloid, a specialized fibrous protein, in diverse tissues, leading to functional impairments.

**Case presentation:**

A 70-year old Asian-Japanese female was referred to our department for further examination of her left hydronephrosis come from lower ureteral obstruction. Contrast enhanced CT and retrograde pyelo-nephrography revealed left ureteral tumor. Though ureteroscropic biopsy did not show malignant pathological findings, ureteroscopic image suspected malignant disease, thus nephroureterectomy was performed. Pathological findings revealed localized ureteral amyloidosis. Whole body examination including gastro endoscopy and cardio ultrasonography could not reveal amyloidosis except ureter. She was free from recurrence 9 months postoperatively.

**Conclusion:**

We herein report a rare case of localized ureteral amyloidosis.

## Introduction

Amyloidosis is a collection of disorders characterized by the extracellular deposition of amyloid, a specialized fibrous protein, in diverse tissues, leading to functional impairments. It encompasses two types: systemic amyloidosis and focal amyloidosis. The prognosis for localized focal amyloidosis is generally positive. Focal amyloidosis represents a mere 2.8% of all amyloidosis cases and is exceedingly rare in the ureter [1–3]. Here, we present a case report of primary amyloidosis affecting the ureter.

## Case presentation

The patient is a 70-year-old Asian-Japanese woman. No special note in her medical history. In October 2018, she visited a house clinic at her local hospital for frequent urination and dysuria, and left hydronephrosis was noted by ultrasound. She then underwent intravenous urography, which revealed left lower ureteral stenosis, and was referred to our department for further examination. Blood and urinary analysis showed no remarkable findings and urinary cytology showed class II.

**Fig. 1:**
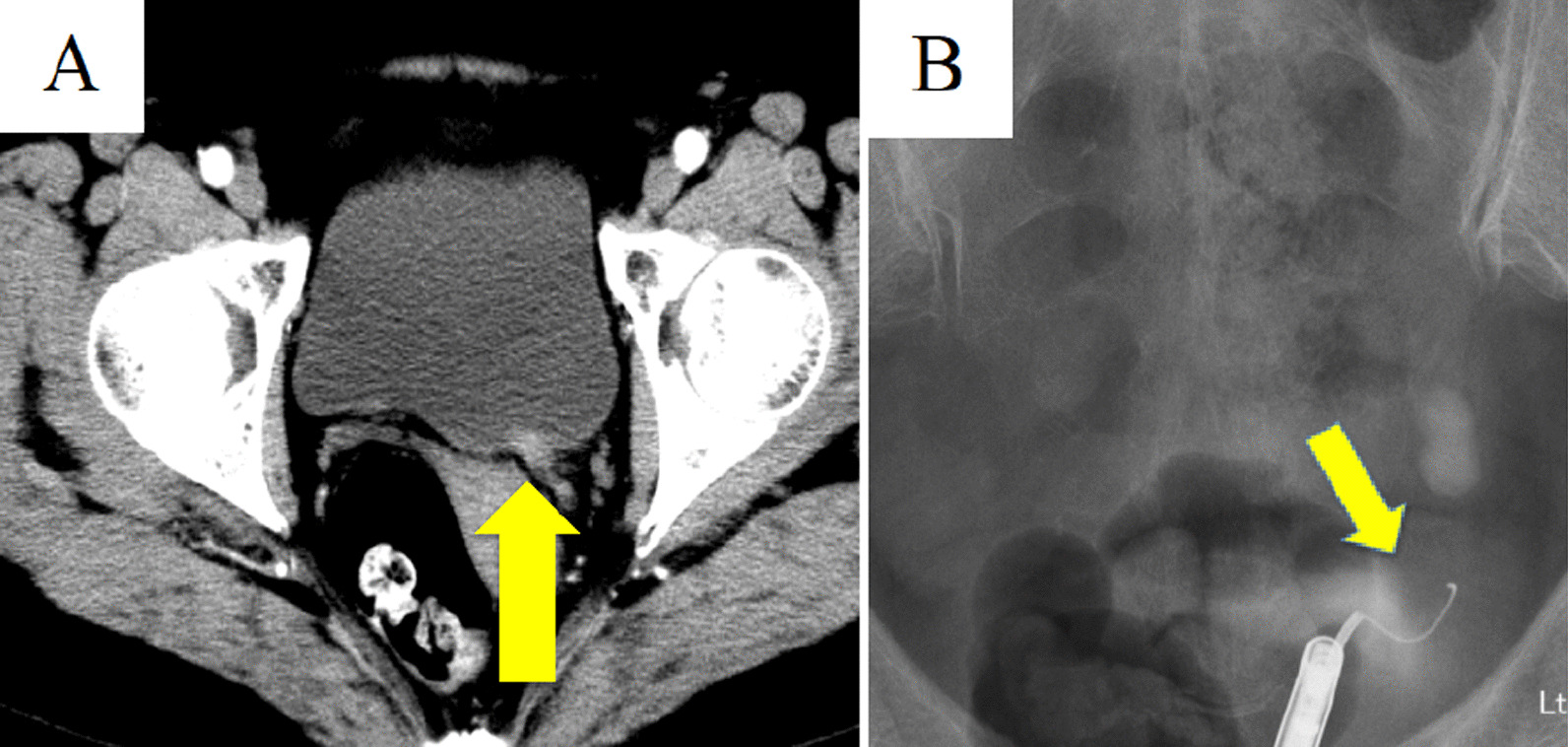
**a** Enhanced computed tomography showed enhanced thick wall in the lower ureter (tumor: arrow). **b** Left lower ureter was completely stenosed in retrograde pyelography (tumor: arrow)

Contrast-enhanced CT showed thickening of the lower ureteral wall with contrast effect (Fig. 1a), and retrograde pyelography showed that the guidewire could not pass through the left ureter due to stenosis of the left lower ureter (Fig. 1b).

Based on the above, a left lower ureteral tumor was suspected, and a left ureteroscopic biopsy was performed in November 2018. Intraoperative findings showed a papillary tumor in the left lower ureter, and four biopsies were performed. Pathological examination showed no malignant findings as regenerative atypia with inflammatory cells. Intraoperative urine cytology showed class III left renal pelvis urine and class I left ureteral urine. Because of the ureteroscopic findings of papillary tumor and insufficient sample volume, ureteral carcinoma could not be ruled out, and a total resection of the left renal pelvis and ureter was performed in January 2019.

**Fig. 2:**
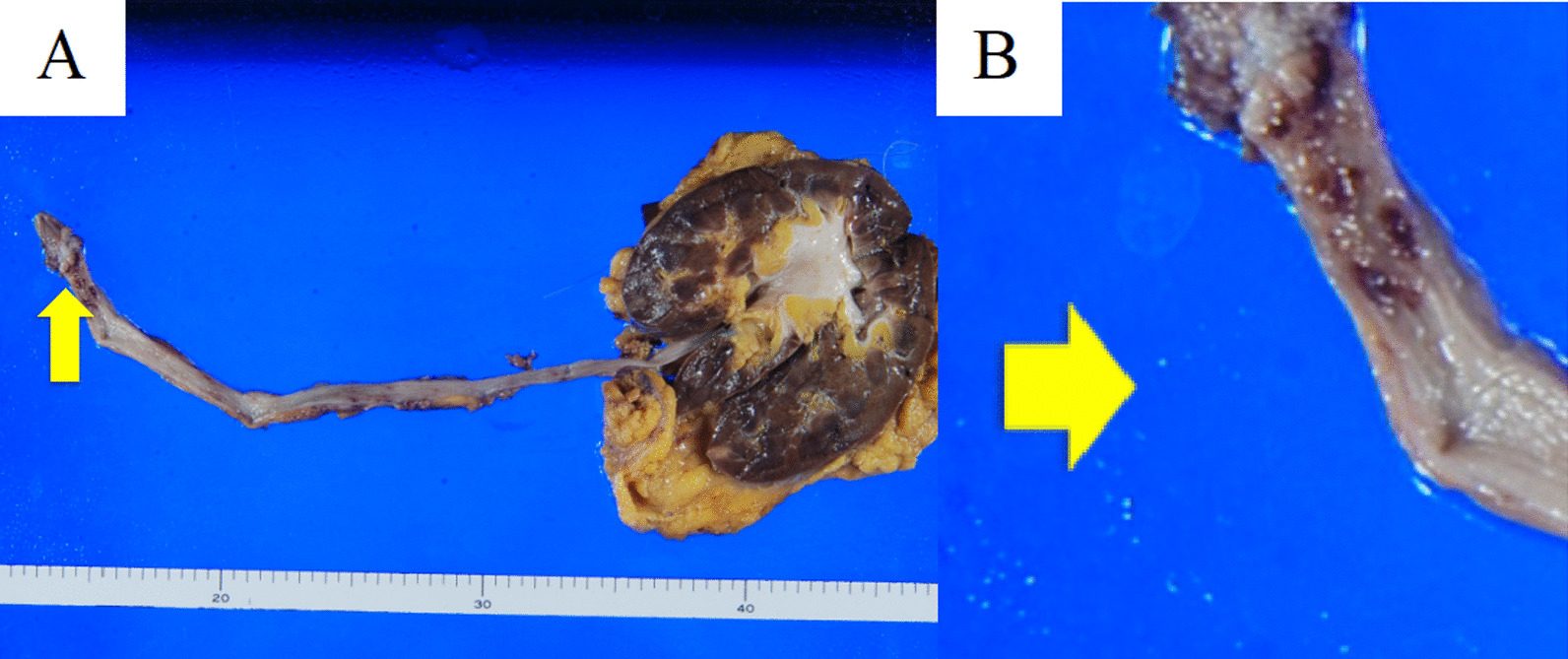
**a** The tumor was founded in lower ureter with 15 × 12 mm size (tumor: arrow). **b** Resected tumor (tumor: arrow)

**Fig. 3:**
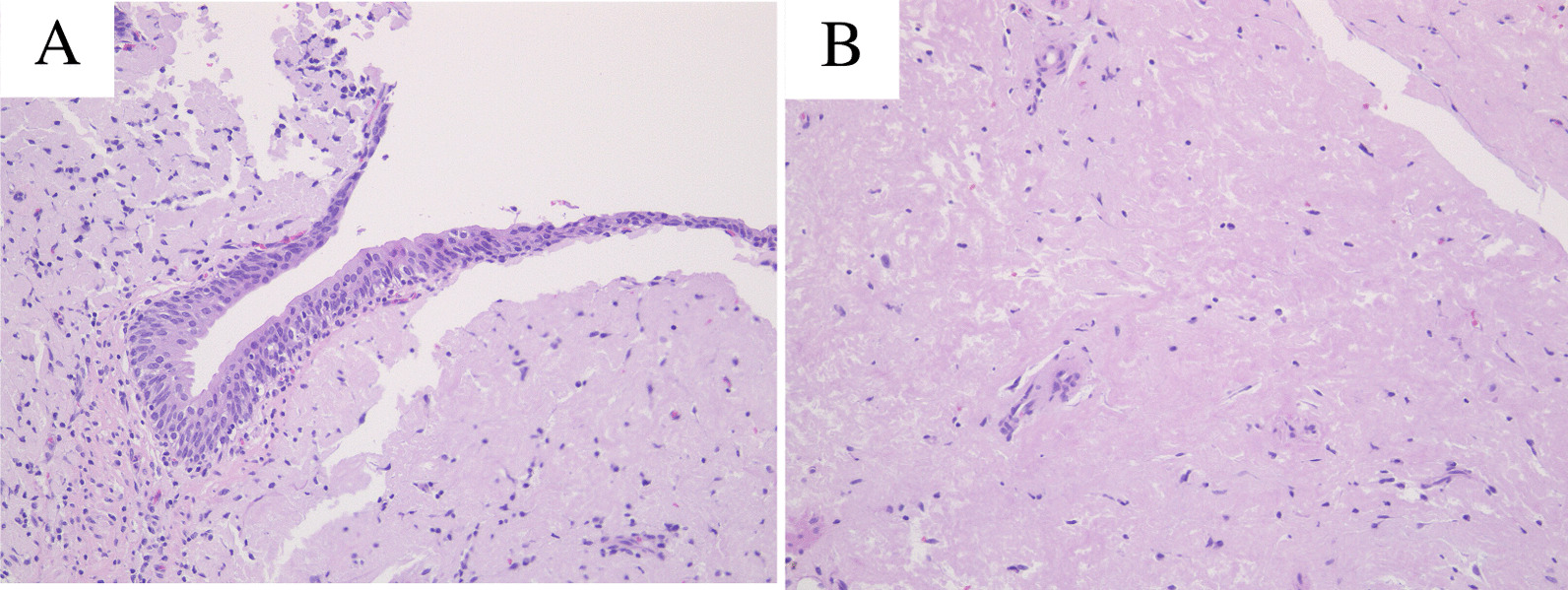
**a** Epithelium shows nuclear enlargement in some areas, but no chromatin hyperplasia or fission pattern. **b** Mildly eosinophilic nonstructural spreading in the interstitium, suspicious of amyloid deposition

**Fig. 4:**
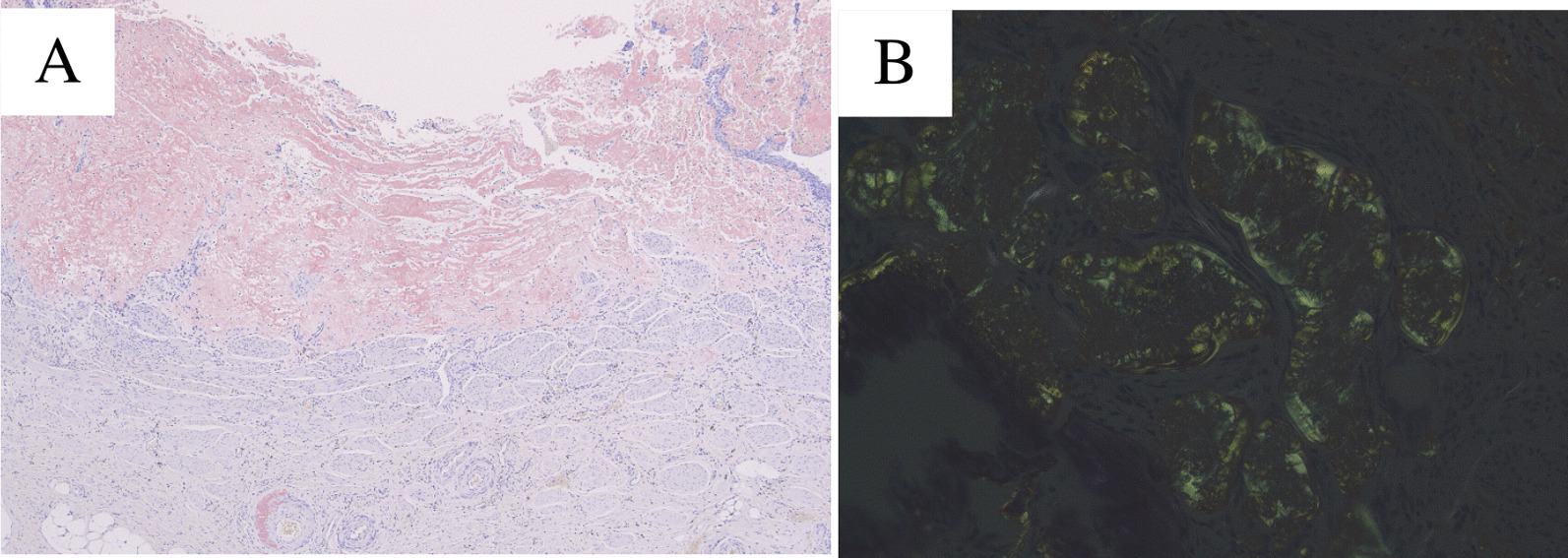
**a** Positive of direct fast scarlet stain (orange) and **b** confocal microscope showed positive (green)

Pathological gross findings revealed a 15 × 12 mm papillary tumor in the left lower ureter (Fig. 2). Histopathologically, HE staining showed no evidence of malignancy in the urothelial cells without chromatin hyperplasia or nuclear fission (Fig. 3a). On the other hand, the interstitium was covered with mildly acidic nonstructural material (Fig. 3b), and since amyloid deposition was suspected, DFS staining and polarized light microscopy were performed, both of which were positive, leading to the diagnosis of ureteral amyloidosis (Fig. 4). No amyloid deposits were found in the excised renal specimen.

**Fig. 5:**
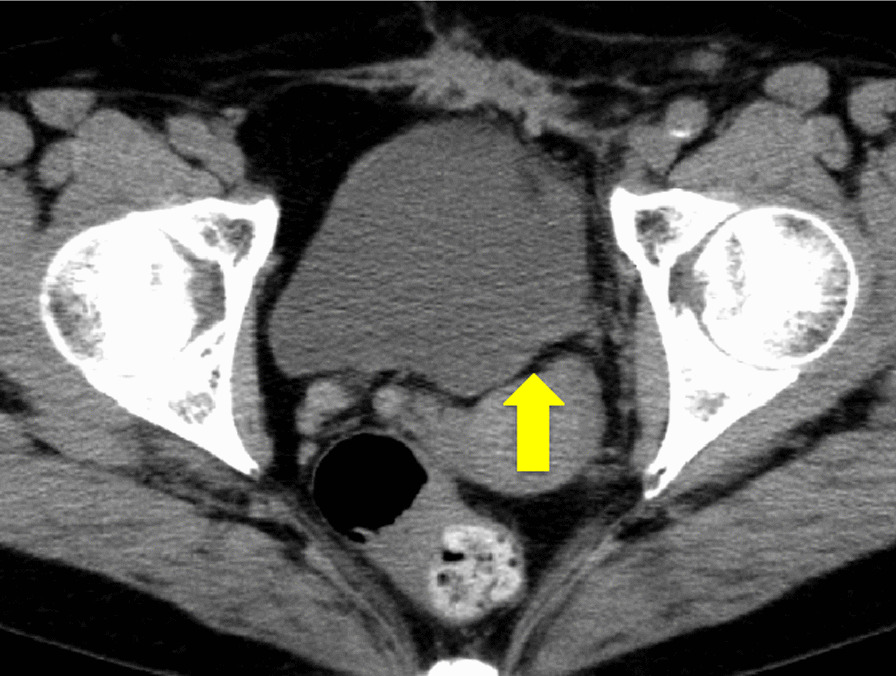
Postoperative computed tomography showed no recurrence (tumor: arrow)

Postoperatively, a systemic search was performed for amyloid deposits. Blood and urine tests showed no abnormal findings and negative urinary Bence-Jones protein. Cardio ultrasound revealed no wall thickening suspicious of amyloid deposition, and upper and lower gastrointestinal endoscopy revealed no findings suggestive of amyloidosis. The patient's postoperative course is good with no recurrence for 9 months after surgery by cystoscopy, ultrasonography, and CT (Fig. 5).

## Discussion

Amyloidosis is a group of diseases in which amyloid, a unique protein with a fibrous structure, is deposited extracellularly in various tissues, causing dysfunction. There are two types of amyloidosis: systemic amyloidosis, which is life-threatening, and localized amyloidosis, which is mostly benign. There are four subtypes of localized amyloidosis, depending on the organ in which the amyloid is deposited. One of these is localized nodular amyloidosis, in which nodular amyloid deposits are found in the respiratory tract, gastrointestinal tract, and urinary tract. Although the definitive diagnosis of localized amyloidosis requires confirmation of the absence of amyloid deposits in other organs, Malek et al. suggest that no further search is necessary unless secondary amyloidosis is ruled out, Bence-Jones protein is negative, serum protein fraction is normal, and rectal biopsy shows no abnormalities [1].

Amyloidosis is a group of diseases in which amyloid, a unique protein with a fibrous structure, is deposited extracellularly in various tissues, causing dysfunction. There are two types of amyloidosis: systemic amyloidosis, which is life-threatening, and localized amyloidosis, which is mostly benign. There are four subtypes of localized amyloidosis, depending on the organ in which the amyloid is deposited. One of these is localized nodular amyloidosis, in which nodular amyloid deposits are found in the respiratory tract, gastrointestinal tract, and urinary tract. The definitive diagnosis of localized amyloidosis requires confirmation of the absence of amyloid deposits in other organs. Malek et al. showed no further search is considered necessary unless secondary amyloidosis is ruled out including Bence-Jones protein, serum protein fraction, and a rectal biopsy [1].

Generally, localized amyloidosis accounts for only 2.8% of all amyloidoses, and those involving the urinary tract are considered to be rare, occurring in the renal pelvis, ureters, bladder, prostate, seminal vesicle gland, seminal duct, testicles, urethra, and penis, with over half of cases being bladder amyloidosis and 25% being ureteral amyloidosis [2, 3].

This case is the 80th case of ureteral amyloidosis reported in Japan. Previous reports showed that the most common chief complaint was hematuria (36 cases), followed by lateral abdominal pain (20 cases), hydronephrosis (9 cases), and lumbar back pain (6 cases). The lower ureter was the most common lesion in 40 cases. The most common treatment was nephroureterectomy in 37 cases, followed by partial ureteral resection in 28 cases and treatment with dimethyl sulfoxide (DMSO) (oral in 2 cases, ODT in 3 cases, and intravesical injection therapy in 4 cases [4]. Ectopic recurrence was reported in one case in the contralateral ureter and one in the vesicoureteral transition zone, but otherwise there were no recurrences, and the prognosis was good. There were no reports of follow-up until death as an outcome.

Preoperative diagnosis of ureteral amyloidosis is difficult, and total nephroureterectomy is often performed because wall thickening and nodular lesions in the urinary tract on CT and hematuria findings do not rule out a malignant tumor in the ureter. Okuda et al. reported that 28 of 42 patients with ureteral amyloidosis in whom a ureteral tumor was suspected preoperatively underwent total nephroureterectomy [5]. In patients with this disease, which is considered to have a good prognosis, it is preferable to choose a treatment that preserves the kidney as much as possible, and partial ureteral resection is performed in many cases in which the diagnosis of this disease can be made by biopsy [5].

The histological site of amyloid deposition in this disease is not clear. Upper gastrointestinal amyloidosis is characterized by amyloid deposition in deeper layers than the epithelium, such as the submucosa and intrinsic muscularis. In previous reports of ureteral amyloidosis, amyloid deposition was often submucosal [6–8]. In the present case, amyloid was deposited in the subcutaneous layer of the urothelium, suggesting a similar pattern of amyloid deposition to that in the gastrointestinal tract. Therefore, biopsy of the ureteral wall thickening and nodular lesions that lack malignant findings may be performed to evaluate the submucosal tissue pathologically to diagnose this disease and to select a treatment that preserves the kidney. In the present case, when the ureteroscopic biopsy lesion was reviewed after a definitive diagnosis of ureteral amyloidosis was made, HE staining of the biopsy specimen revealed acidophilic, unstructured material that appeared to be amyloid deposition.

Although no certain opinion has been obtained on the method and duration of follow-up, there have been reports of ectopic recurrence and complications of urothelial carcinoma, so routine anually follow-up with CT and ultrasound are planned [9].

## Conclusion

We herein report a rare case of ureteral amyloidosis.

## Data Availability

Due to ethical restrictions, the raw data underlying this paper are available upon request to the corresponding author.
